# MSA U-Net: a multi-scale attention-enhanced network for automatic segmentation of uterine lymph nodes in MRI images

**DOI:** 10.1186/s43046-026-00391-6

**Published:** 2026-08-03

**Authors:** Jingde Hong, Chunxia Chen, Juping Qiu, Zeng Lin, Yuhang Xie, Yongping Lin

**Affiliations:** 1 School of Optoelectronic and Communication Engineering, Xiamen University of Technology, Amoy, China; 2 Department of Radiology, Fujian Maternal and Child Hospital, Fuzhou, China; 3 Fujian Key Laboratory of Women and Children’s Critical Diseases Research, Fuzhou, China

**Keywords:** Lymph nodes, Multi-scale attention, Magnetic resonance imaging, Automatic segmentation

## Abstract

**Objective:**

Lymph nodes (LNs) in medical images often have fuzzy boundaries, vary in size and shape, and have intensities similar to those of neighboring tissues, making accurate segmentation challenging. To address this issue, we propose a multi-scale attention-enhanced network (MSA U-Net) that integrates channel-wise and spatial attention mechanisms for automatic segmentation of metastatic pelvic LNs associated with uterine malignancies from sagittal magnetic resonance imaging (MRI).

**Methods:**

This network integrates a U-Net backbone, fuses features from different encoder levels at skip connections, and feeds the fused features into the decoder at each layer. Different receptive fields are used at the bottleneck nodes to capture multiscale contextual information. A detection head is added to the bottom layer of the network and the detection results are used to assist in the final segmentation of the image. A lightweight design of the convolutional block attention module is also implemented to optimize feature representation.

**Results:**

The experimental results demonstrate that the proposed network achieves better segmentation performance compared to the baseline model. The proposed network achieves a mean intersection over union of 0.76, an average pixel accuracy of 0.97, a precision of 0.78, a recall of 0.97, a Dice coefficient of 0.82, and a Hausdorff distance of 2.21.

**Conclusions:**

The proposed MSA U-Net segmentation network effectively segments LNs in uterine MRI images, outperforming existing segmentation methods. This study provides a reliable and automated method to help clinicians detect LN, thus improving clinical decision-making.

## Introduction

Accurate segmentation of medical images is a crucial step in computer-aided diagnosis, enabling quantitative analysis and interpretation of anatomical and pathological information [1]. This process accelerates clinical workflows, reduces the risk of human error, and ultimately improves diagnostic precision and patient outcomes [2]. Among various segmentation techniques, encoder–decoder architectures have become a widely adopted framework for semantic segmentation due to their hierarchical design, which facilitates the progressive capture and reconstruction of spatial and contextual information [3–6]. However, repeated pooling and convolution operations in the encoder can lead to the loss of fine-grained spatial details, posing challenges for accurately delineating small or low-contrast anatomical structures.

Building upon the encoder–decoder paradigm, convolutional neural networks (CNNs) have achieved remarkable success in both computer vision [7–9] and medical image analysis [10, 11]. In particular, accurate detection and segmentation of lymph nodes (LNs) are essential for cancer diagnosis, staging, and treatment planning. In patients with uterine malignancies, the presence and extent of pelvic LN involvement constitute important imaging biomarkers for disease staging, treatment planning, and prognosis assessment. Numerous CNN-based methods have been developed to analyze mediastinal, abdominal, pelvic, and cervical LNs [12]. For example, Roth et al. [13] proposed a 2.5D CNN with multi-view resampling for robust LN classification on CT, while Zhang et al. [14] introduced an mpMRI-based detection and segmentation model (auto-LNDS) using Mask R-CNN, which outperformed junior radiologists. Similarly, Mlynarski et al. [15] designed a 3D patch-based CNN that achieved high localization accuracy for LN staging, and Wang et al. [16] developed a multi-module AI system (LNAS) for chest CT, demonstrating strong generalization across large-scale datasets. Despite these advances, LN segmentation remains challenging due to the small size, indistinct boundaries, and variable appearance of LNs across patients and imaging modalities. The conventional U-Net architecture, though widely adopted, often loses crucial spatial information through repeated downsampling, resulting in suboptimal segmentation of small or thin structures.

To overcome these limitations, recent research has sought to enhance U-Net–based architectures through improved feature representation and multi-scale information fusion. U-Net++ [17] and U-Net 3+ [18] enhance feature aggregation via dense or full-scale skip connections, while recurrent and residual variants such as RU-Net and R2U-Net [19] strengthen representation through iterative refinement. MultiResUNet [20] and BCDU-Net [21] further integrate multi-resolution and bidirectional feature propagation to improve segmentation robustness. However, these extensions typically increase computational cost and memory usage, which can limit clinical deployment. In parallel, multi-scale fusion strategies have been proposed to better capture contextual dependencies. DeepLabv3+ [22] combines atrous spatial pyramid pooling with a lightweight decoder to extract semantic context at multiple scales, while DMCGNet [23], FCP-Net [24], and NMNet [25] employ adaptive feature fusion and cross-scale attention to enhance hierarchical representation. Recent studies have further demonstrated the effectiveness of multi-scale representation learning in medical image analysis. Agarwal et al. [26] proposed a multi-scale dual-channel decoder that jointly captures short- and long-range dependencies through hierarchical feature embedding, while Gong et al. [27] introduced a multi-scale feature fusion strategy with adaptive channel selection to improve the detection of small pelvic lymph node metastases. Similarly, Datta et al. [28] integrated multi-scale pooling and attention mechanisms within a Swin-UNETR framework to enhance tumor segmentation performance. These studies collectively demonstrate that effective multi-scale feature aggregation plays a critical role in preserving contextual information and improving segmentation accuracy. However, many of these approaches rely on transformer-based architectures or complex feature fusion schemes, resulting in increased computational burden and deployment difficulty.

More recently, attention mechanisms have been introduced to adaptively emphasize informative regions while suppressing irrelevant background. Attention U-Net [29] introduced attention gates to refine skip-connected features, leading to more precise delineation of target structures. Building on this idea, CA-Net [30], MsTGANet [31], and CFNet [32] combine spatial, channel, and scale attention for improved segmentation accuracy across modalities. Wang et al. [33] further proposed a cross-scale attention-guided U-Net that embeds transformer-based cross-attention modules into skip connections to enhance global–local feature interaction, while Liu et al. [34] incorporated attention mechanisms into a cross-domain synthesis and segmentation framework to improve boundary delineation and anatomical consistency. In addition, Lin et al. [35] demonstrated that lightweight attention mechanisms can effectively emphasize diagnostically important regions while maintaining computational efficiency in MRI analysis tasks. Although these methods improve model focus and feature discrimination, they often introduce additional computational complexity or rely on transformer-based designs. To address this issue, lightweight attention modules such as the convolutional block attention module (CBAM) [36] have been proposed to achieve effective feature refinement with minimal parameter overhead.

Despite these advances, several limitations remain. Existing attention-based U-Net variants primarily focus on feature refinement and often lack explicit mechanisms for capturing multi-scale contextual information or providing localization guidance for small targets. Conversely, multi-scale U-Net architectures enhance receptive-field diversity but generally treat all regions equally and may struggle to suppress irrelevant background responses. Detection-assisted segmentation frameworks improve object localization; however, detection and segmentation are often implemented as loosely coupled components, limiting their ability to jointly optimize feature representation and boundary delineation. Consequently, effectively integrating multi-scale representation learning, discriminative feature refinement, and localization guidance within a unified framework remains an open challenge for small LN segmentation.

Motivated by these developments, we observe that existing studies typically focus on improving segmentation performance from a single perspective, such as multi-scale feature aggregation, attention-guided feature refinement, or detection-assisted localization. However, uterine LN segmentation remains particularly challenging because LN are often small, exhibit weak contrast against surrounding tissues, and require both accurate localization and precise boundary delineation. Simply introducing an individual module is often insufficient to simultaneously address these challenges.

To address the above limitations, we propose MSA U-Net, a unified framework that synergistically integrates multi-scale feature fusion, lightweight attention refinement, and detection-guided localization within a U-Net architecture. The proposed framework explicitly incorporates multi-scale contextual aggregation through both skip-connection fusion and a multi-branch dilated bottleneck, thereby extending conventional attention U-Net designs that mainly emphasize feature selection. It also introduces lightweight attention refinement to selectively enhance discriminative LN features while suppressing irrelevant background responses, which complements existing multi-scale architectures that primarily focus on feature representation. In addition, the detection branch is embedded within the segmentation framework to provide auxiliary localization cues that directly participate in segmentation refinement, rather than being loosely coupled with the segmentation process as in conventional detection-assisted approaches. Through this collaborative design, the proposed framework aims to improve both localization accuracy and boundary delineation of small LN while maintaining practical computational efficiency.

The main contributions of this study are summarized as follows:

- A lightweight attention mechanism is incorporated at each decoding stage to selectively enhance informative LN features and suppress irrelevant background responses while maintaining computational efficiency.
- A multi-branch bottleneck and multilevel feature fusion strategy are developed to capture complementary contextual information across different receptive fields and improve the representation of small LN.
- A unified detection-guided segmentation framework is developed to jointly exploit multi-scale contextual learning, lightweight attention refinement, and auxiliary localization cues, enabling collaborative optimization of localization and segmentation for small LN.

## Methods

### Materials

This study utilized a retrospective dataset collected from the Fujian Provincial Maternity and Child Health Hospital, with ethical approval obtained from the Institutional Review Board and a waiver of informed consent due to its retrospective nature. In this study, the term “uterine lymph nodes (LNs)” refers to pelvic LNs identified on MRI examinations of patients with uterine malignancies. All included cases were pathologically confirmed to have LN metastasis, and the annotated targets therefore correspond to metastatic pelvic LNs associated with uterine tumors. Initially, 165 patients who had undergone uterine LN MRI scans were identified through the hospital’s PACS system. After applying exclusion criteria, including absence of pathological confirmation, misalignment with LN stage III classification, and missing axial T2-weighted imaging (T2WI) or diffusion-weighted MRI sequences, a total of 123 eligible cases (mean age: 53.4 ± 8.5 years) remained for analysis. All cases included were pathologically verified.

**Fig. 1:**
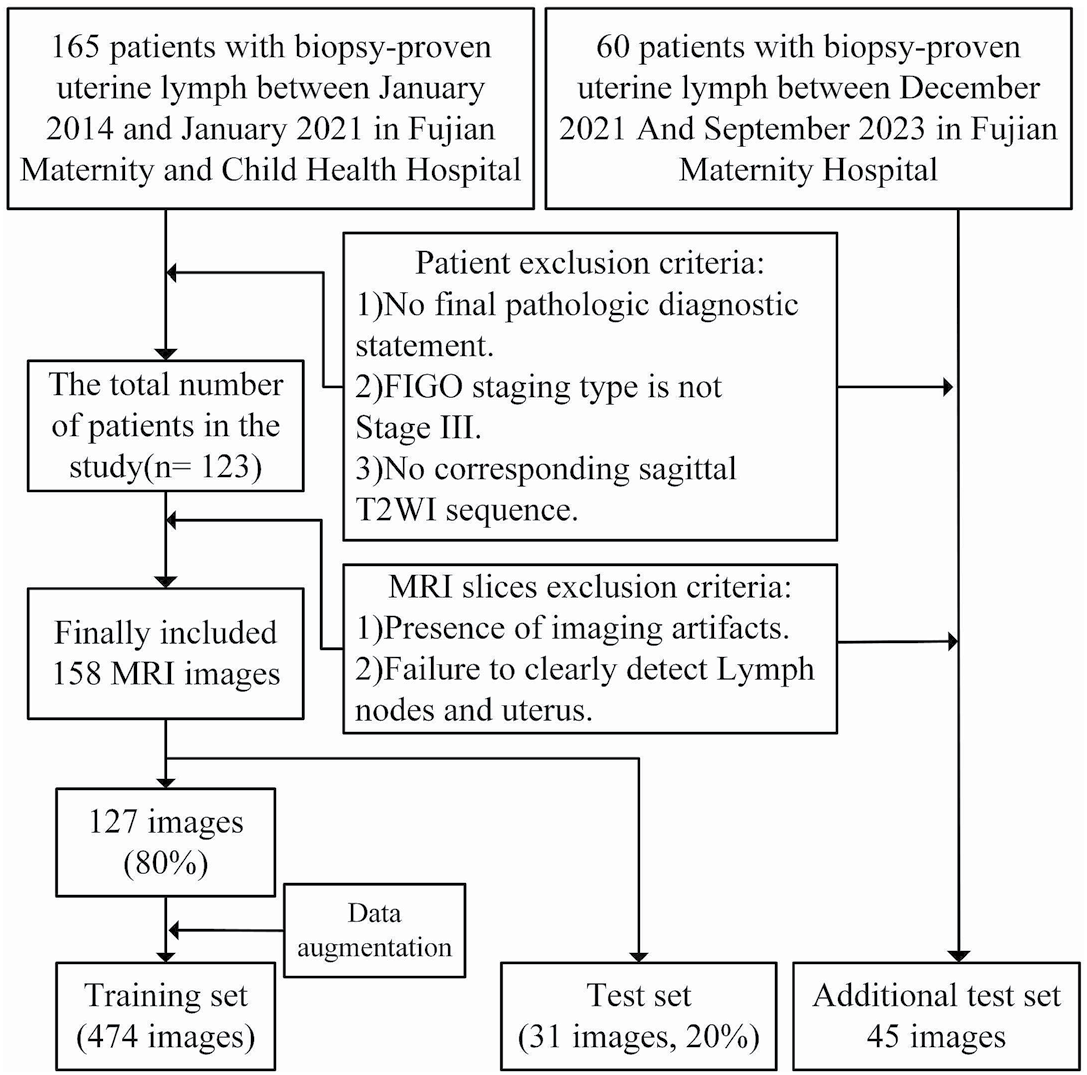
The process of constructing the dataset. It includes data sources, exclusion criteria, and dataset partitioning

Following this, the axial T2WI scans were manually reviewed and annotated by two experienced radiologists with more than 10 years of experience in pelvic MRI interpretation (the screening process is shown in Fig. 1). LN regions were delineated using LabelMe (version 4.5.7) according to standardized annotation criteria established by the institution. The initial annotations were performed by one radiologist and subsequently reviewed by a second radiologist. In cases of disagreement, a consensus was reached through joint discussion to ensure annotation consistency and reliability. The final consensus annotations were used as the reference standard for model training and evaluation. The resulting dataset comprises 158 MRI images, as multiple representative axial T2WI slices covering the LNs regions were selected from some patients. Each slice was treated as an independent sample for the segmentation task. The dataset was split into a training cohort (127 images) and a test cohort (31 images), ensuring consistent quality and diagnostic value across samples. Importantly, data splitting was performed at the patient level, and images from the same patient were strictly assigned to only one subset (either training or testing) to prevent data leakage. To mitigate the limitations imposed by the small dataset size, a robust data augmentation pipeline was employed using the imgaug library. The augmentation procedures included random flipping (horizontal and vertical), slight rotation within $\pm 15^\circ$, application of Gaussian blur and noise, linear contrast enhancement, and adjustments in hue and saturation. Each sample in the training set was augmented multiple times to generate a more diverse dataset, resulting in a total of 474 images. These augmentation strategies were adopted to increase data diversity and reduce the risk of model overfitting caused by the limited dataset size.

In addition, to assess generalization performance, an independent test set comprising 45 MRI images from Fujian Maternity Hospital was incorporated, which remained unseen during model training.

### MSA U-net network framework model

**Fig. 2:**
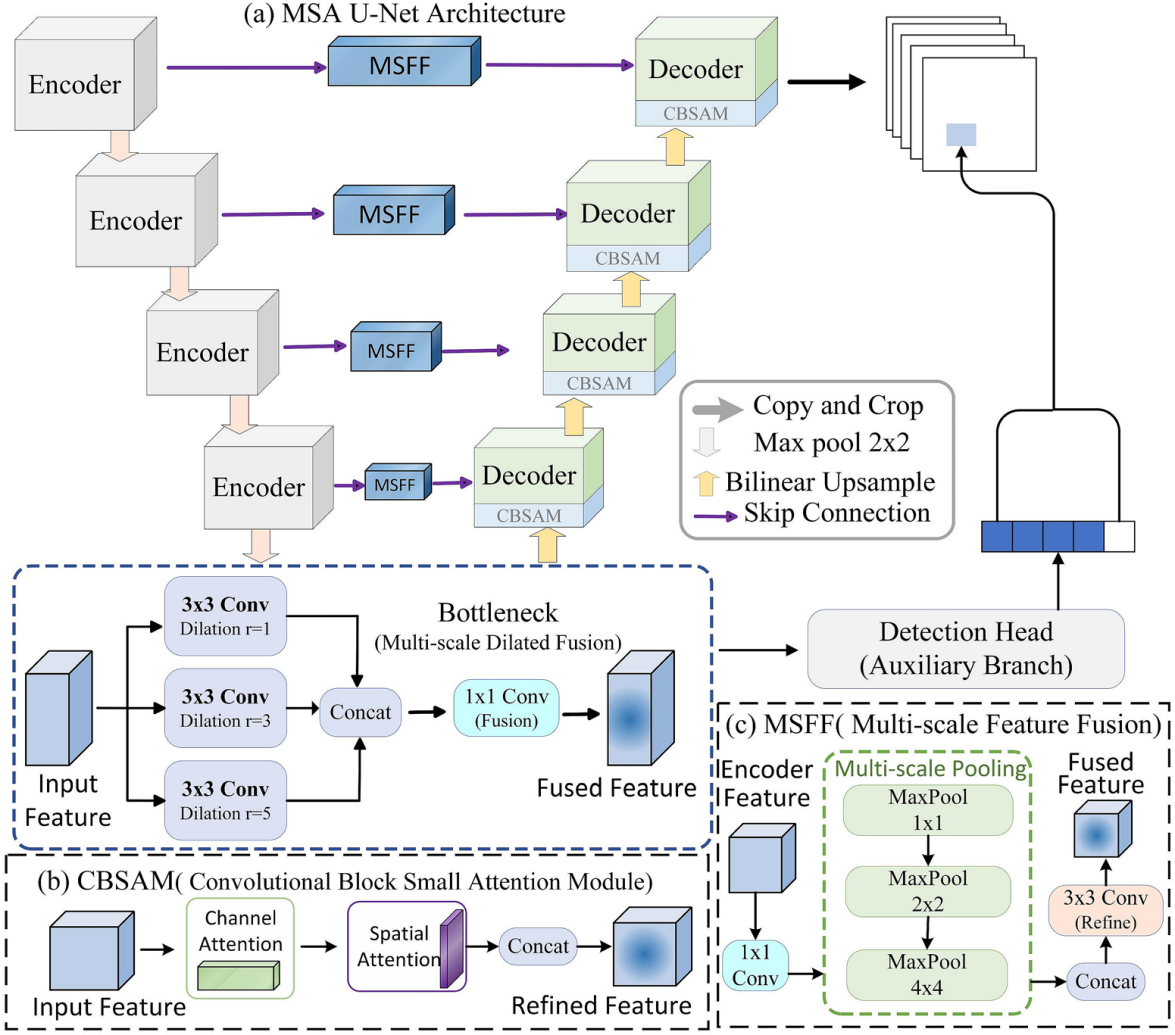
Overview of the proposed MSA U-Net for pelvic metastatic lymph node segmentation in uterine MRI. **a** Overall architecture illustrating the encoder–decoder framework, bottleneck multi-scale dilated fusion, CBSAM refinement, and detection-guided segmentation; **b** detailed structure of the CBSAM module; **c** detailed structure of the multi-scale feature fusion (MSFF) module

To enhance the segmentation performance of pelvic metastatic lymph nodes in uterine MRI images, we proposed MSA U-Net, as illustrated in Fig. 2. The overall framework consists of three complementary components: (1) multi-scale feature fusion through MSFF-enhanced skip connections, (2) CBSAM-based attention refinement together with bottleneck multi-scale dilated fusion for robust feature representation, and (3) a detection-guided localization branch that provides auxiliary localization cues to improve segmentation accuracy. The model retains the classical encoder–decoder framework while incorporating a series of architectural improvements, including multi-scale feature extraction in skip connections, a small-object attention mechanism, and an auxiliary detection branch. Specifically, a multi-scale feature extraction module is embedded in each skip connection between the encoder and decoder, and the extracted multi-scale features are then fused and passed to the corresponding decoder layer, enabling the network to better handle scale variations and preserve fine-grained information. Parallel dilated convolutions with different dilation rates (*r* = 1, 3, 5) are applied at the bottom of the network to capture contextual features from different receptive fields. To further emphasize the discriminative features associated with small targets, a small-object attention module is introduced, which sequentially applies channel and spatial attention to selectively enhance relevant regions and suppress irrelevant background noise. In addition, an auxiliary detection head is incorporated at the bottom of the network to provide additional supervision and improve localization accuracy using deep semantic features. With these enhancements, the proposed network significantly strengthens its ability to detect and segment small, low-contrast structures, making it well suited for automated medical image analysis tasks involving subtle and complex anatomical targets.

To ensure feature consistency across different encoder levels, feature maps from intermediate encoder stages are first spatially aligned using bilinear interpolation before fusion. Specifically, deeper semantic features are resized to a common spatial resolution and concatenated with the corresponding encoder features. A subsequent convolutional refinement operation is then applied to reduce channel redundancy and generate a unified multi-scale representation. The resulting fused feature maps are propagated to the decoder through skip connections, enabling effective integration of low-level spatial details and high-level contextual information for accurate LN segmentation.

#### Multi-scale dilated convolution fusion

At the bottom of the encoder–decoder architecture, we adopt parallel dilated convolutions to capture contextual features from multiple receptive fields. Given the deepest feature map $F_5 \in \mathbb {R}^{C \times H \times W}$, three parallel *3× 3* convolutions with different dilation rates $r \in \{1, 3, 5\}$ are applied:

1 $$\begin{aligned} F_r = f^{3\times 3}_r(F_5), \quad r \in \{1, 3, 5\}, \end{aligned}$$

where $f^{3\times 3}_r(\cdot )$ denotes a *3× 3* convolution with dilation rate *r*. The outputs are concatenated and fused through a *1× 1* convolution to aggregate the multi-scale information:

2 $$\begin{aligned} F_{\text {fusion}} = f^{1\times 1}\!\left( [F_1, F_3, F_5]\right) , \end{aligned}$$

where *[· ]* denotes channel-wise concatenation. This fusion process enriches contextual representations and provides the subsequent detection and segmentation branches with both local and global semantic cues.

#### Convolutional block small attention module

To enhance the network’s ability to focus on small target regions while maintaining computational efficiency, we propose a modified version of the convolutional block small attention module (CBSAM), as illustrated in Fig. 2. Unlike the original CBAM, which sequentially applies channel and spatial attention to all features within a single processing stream, our design explicitly separates and simplifies the two attention branches to reduce computational overhead and emphasize spatial refinement in small-object contexts. In the proposed Small Target Attention Module, the input feature first passes through a lightweight Channel Attention Module, which employs only max pooling (omitting average pooling used in the original CBAM) and a shared multilayer perceptron (MLP) to minimize parameter usage. This is followed by a Spatial Attention Module that operates on the channel-refined features using a single convolution layer to capture local spatial dependencies. The outputs of both modules are then combined through element-wise addition to produce the final refined feature representation.

This design reduces redundant attention computation and introduces more targeted enhancement by decoupling spatial and channel interactions. More importantly, it is tailored to preserve discriminative features of small and low-contrast targets, which are often suppressed in standard CBAM due to global pooling operations. Consequently, the improved attention module achieves a favorable balance between representational precision and model complexity, making it well suited for small-object segmentation tasks in medical image analysis.

Channel Attention. Given an input feature map $X \in \mathbb {R}^{C \times H \times W}$, the channel attention map $M_c(X)$ is computed as:

3 $$\begin{aligned} M_c(X) = \sigma \left( W_2 \,\delta \!\left( W_1 \, \textrm{MaxPool}(X)\right) \right) , \end{aligned}$$

where $\textrm{MaxPool}(\cdot )$ denotes global max pooling, $W_1 \in \mathbb {R}^{\frac{C}{r} \times C}$ and $W_2 \in \mathbb {R}^{C \times \frac{C}{r}}$ are learnable weights, $\delta (\cdot )$ is the ReLU activation, and $\sigma (\cdot )$ is the sigmoid function.

Spatial Attention. To capture local spatial dependencies, a spatial attention map $M_s(X)$ is obtained using a single *1 × 1* convolution:

4 $$\begin{aligned} M_s(X) = \sigma \left( f^{1\times 1}(X)\right) , \end{aligned}$$

where $f^{1\times 1}(\cdot )$ denotes a *1× 1* convolutional operation.

Feature Refinement. The final refined feature is obtained through element-wise multiplication and addition of both attention maps:

5 $$\begin{aligned} Y = X \odot (M_c(X) + M_s(X)), \end{aligned}$$

where *⊙* denotes element-wise multiplication.

Equation 5 introduces an additive fusion strategy instead of the traditional multiplicative one, making the attention mechanism more sensitive to weak and small target regions that might otherwise be suppressed.

#### Object detection module

To enhance the model’s ability for precise lesion localization, particularly in small object contexts, we incorporated a dedicated detection head into the segmentation backbone. This detection branch operates directly on the deepest semantic feature map obtained after multi-scale dilated convolutional fusion, which captures contextual information across multiple receptive fields. The detection head consists of three successive 3*×*3 convolutional layers (each with 256 filters and ReLU activation) that progressively refine the features while maintaining spatial resolution. It then bifurcates into two parallel 1*×*1 convolutional layers: one for object classification (cls_head, outputting per-pixel class scores) and the other for bounding box regression (reg_head, predicting four offset parameters). This structure is computationally efficient yet sufficiently expressive to support robust region-level prediction in parallel with pixel-level segmentation. To maintain semantic consistency between detection and segmentation outputs, the class score map from the detection branch is upsampled and fused with the final segmentation output through two additional convolutional layers. This segmentation–detection fusion enhances boundary precision and improves the detection of small or low-contrast targets, which may be underrepresented in pure segmentation outputs. Overall, this design enables the network to leverage both global region cues and fine-grained pixel-level information, facilitating accurate dual-task learning.

**Fig. 3:**
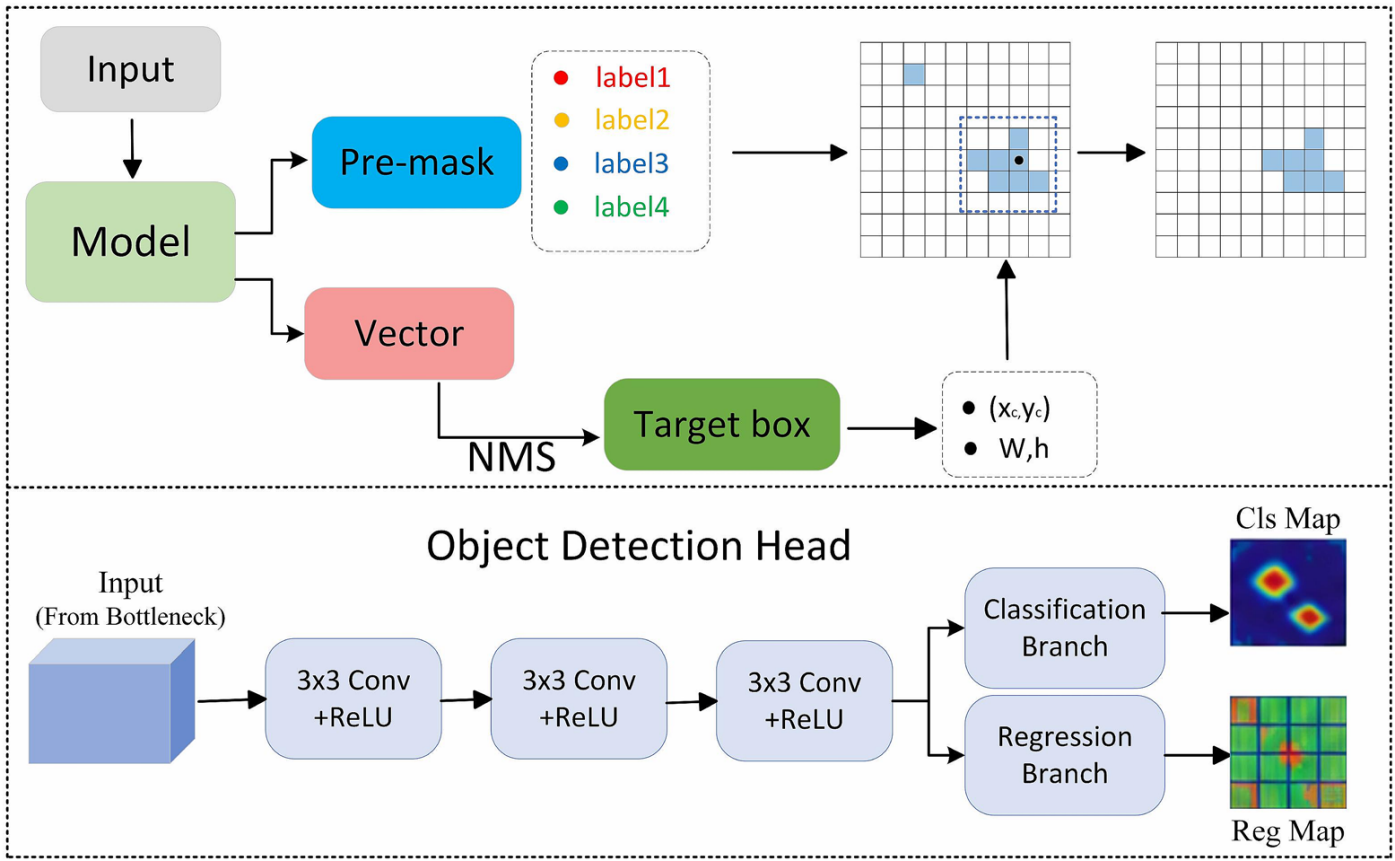
Pipeline of MSA U-Net with detection head

The overall prediction pipeline of the proposed framework is illustrated in Fig. 3. Given an input MRI slice, the model produces two parallel outputs: a preliminary segmentation mask generated by the decoder path and detection vectors produced by the dedicated detection branch. The detection head exploits deep semantic features and predicts both classification scores and bounding box regression vectors, where each vector encodes the target’s center coordinates $(x_c, y_c)$ along with its width and height (*w*, *h*). Thus, the detection branch produces two complementary outputs: a class probability map indicating the likelihood of LN presence and a bounding-box regression map encoding localization offsets for each spatial location. Non-maximum suppression (NMS) is subsequently applied to refine the predicted bounding boxes, which serve as coarse localization priors. These bounding boxes are then mapped onto the Pre-mask, spatially constraining the segmentation and guiding the refinement process. By suppressing irrelevant false positives outside the detected regions, the model ensures more precise delineation of LNs. As illustrated in the figure, this joint mechanism combines region-level localization from bounding box regression with pixel-level refinement from the segmentation mask, thereby improving boundary accuracy and enhancing robustness in detecting small or low-contrast LNs in uterine MRI scans.

Let $F_{\text {fusion}}$ denote the multi-scale fused feature map. The detection head first refines it through three consecutive convolutional layers:

6 $$\begin{aligned} F_d = \delta \!\left( f^{3\times 3}_3\!\left( \delta \!\left( f^{3\times 3}_2\!\left( \delta \!\left( f^{3\times 3}_1(F_{\text {fusion}})\right) \right) \right) \right) \right) , \end{aligned}$$

where $\delta (\cdot )$ denotes the ReLU activation. Then, two parallel prediction branches are applied:

7 $$\begin{aligned} P_{\text {cls}}= & f^{1\times 1}_{\text {cls}}(F_d), \end{aligned}$$

8 $$\begin{aligned} P_{\text {reg}}= & f^{1\times 1}_{\text {reg}}(F_d), \end{aligned}$$

where $P_{\text {cls}} \in \mathbb {R}^{C \times H \times W}$ denotes the dense class probability map generated by the detection branch, and $P_{\text {reg}} \in \mathbb {R}^{4 \times H \times W}$ represents the corresponding bounding-box regression map, which encodes localization offsets $(\Delta x,\Delta y,\Delta w,\Delta h)$ relative to each spatial position. Together, these outputs provide coarse region-level localization cues that complement the pixel-level segmentation predictions.

Segmentation–Detection Fusion. Finally, the detection branch output is fused with the segmentation result to enhance consistency between the two tasks:

9 $$\begin{aligned} S_{\text {fused}} = f^{1\times 1}_2\!\left( f^{3\times 3}_1([S, \hat{P}_{\text {cls}}])\right) , \end{aligned}$$

where *S* denotes the decoder-generated segmentation mask and $\hat{P}_{\text {cls}}$ is the upsampled detection score map. This operation integrates region-level cues from detection with pixel-level segmentation features to yield refined, anatomically coherent predictions.

## Experiments and results

### Training process

All experiments were conducted on a workstation running Windows 10. The proposed model was implemented using Python 3.10.18 and PyTorch 2.3.0, with TorchVision 0.18.0. Model training was accelerated using CUDA 12.1 and cuDNN 8.8 on an NVIDIA GeForce RTX 4090 D GPU with 24 GB memory. Additional libraries, including NumPy, SciPy, OpenCV, timm, and einops, were employed for data processing, augmentation, and model implementation.

All models mentioned in this work were optimized using the Adam optimizer. For a fair comparison, all competing segmentation models, including U-Net, U-Net++, Attention U-Net, and ResUNet, were trained under the same experimental environment, dataset partition, optimizer settings, batch size, learning rate schedule, and stopping criteria whenever applicable. Hyperparameter selection followed the recommendations reported in the corresponding original studies and was further verified through preliminary validation experiments to ensure a fair and consistent comparison.

The initial hyperparameter settings were first determined empirically according to commonly adopted configurations in medical image segmentation studies and our prior experimental experience. Subsequently, a series of preliminary validation experiments were conducted to investigate different learning rates (1e-2, 1e-3, and 1e-4) and weight decay settings. Based on the convergence behavior and validation performance, an initial learning rate of 1e-3 and a weight decay of 5e-4 were selected as the final training parameters. Early stopping was adopted to prevent overfitting, and training was terminated when no performance improvement was observed within the last 100 epochs. To facilitate network optimization and improve feature generalization, the backbone was initialized using weights pretrained on the PASCAL VOC dataset.

Under the above configuration, training of the proposed MSA U-Net required approximately 1.5 hours to complete on a single NVIDIA RTX 4090 D GPU.

### Evaluation index

In this study, multiple commonly used metrics were employed to evaluate segmentation performance, including intersection over union (IoU), mean IoU (mIoU), pixel accuracy (PA), mean pixel accuracy (mPA), Precision, Recall, and the Dice coefficient, were used to assess the overlap and pixel-wise classification accuracy between the predicted segmentation and the ground truth. These metrics are widely adopted in medical image segmentation and therefore are not elaborated in detail here.

In addition, the hausdorff distance (HD) was used to quantify boundary similarity between the predicted segmentation mask *X* and the ground truth mask *Y*. HD measures the maximum distance between the boundary points of two sets and is particularly suitable for assessing boundary accuracy in small-structure segmentation tasks. It is defined as follows:

10 $$\begin{aligned} {HD} (X, Y) = \max \left\{ \sup _{x \in X} \inf _{y \in Y} d(x,y), \; \sup _{y \in Y} \inf _{x \in X} d(x,y) \right\} \end{aligned}$$

where *d(x,y)* is the Euclidean distance between a point *x ∈ X* and a point *y ∈ Y*, *\sup* denotes the supremum, and *∈f* denotes the infimum. A lower HD indicates better boundary alignment.

### Results

To validate the effectiveness of each proposed module in MSA U-Net, we conducted a series of ablation experiments. The baseline U-Net achieved an mIoU of 0.64 and a Dice coefficient of 0.68. Incorporating multi-scale feature extraction into the skip connections improved the mIoU to 0.75 and Dice to 0.71, demonstrating the benefit of enhancing feature propagation between the encoder and decoder. Adding multi-scale feature extraction to the encoder alone increased the mIoU to 0.74 and Dice to 0.76, indicating that richer contextual representations in the encoder can also improve segmentation performance. Combining both strategies further improved the overall performance, yielding an mIoU of 0.73 and a Dice coefficient of 0.74.

The introduction of the auxiliary detection head further enhanced localization capability, achieving an mIoU of 0.74, a Dice coefficient of 0.78, and a recall of 0.95. Finally, integrating the small-object attention mechanism achieved the best overall segmentation performance, with an mIoU of 0.88, mPA of 0.93, precision of 0.94, recall of 0.93, Dice coefficient of 0.94, and HD of 2.01, demonstrating its effectiveness in improving both lesion localization and boundary delineation. These results indicate that each proposed module contributes positively to segmentation performance, while the complete MSA U-Net achieves the best overall performance through the integration of all proposed components (Table 1).

To assess the statistical significance of these improvements, we conducted paired t-tests comparing the full model with models excluding individual modules. As summarized in Table 2, removing any key module (Combination, Detection box, or Attention) led to significant decreases in mIoU, Dice, and other performance metrics, accompanied by corresponding increases in HD (*p<0.05*). Notably, omission of the Attention module resulted in the largest increase in HD (0.005), underscoring its critical role in refining small-object boundaries. These findings confirm that all proposed modules contribute significantly to segmentation performance, and their integration is essential for accurate and robust LN segmentation in uterine MRI images.

**Table 1:** Ablation experiments on the internal test set. Skip connection denotes adding multi-scale feature extraction to the skip connections, Encoder denotes adding multi-scale feature extraction to the encoder, and Combination denotes integrating both

U-Net	Skip connection	Encoder	Combination	Detect box	Attention	mIoU	mPA	Precision	Recall	Dice	HD
*\checkmark*						0.64	0.69	0.74	0.84	0.68	2.56
*\checkmark*	*\checkmark*					0.75	0.82	0.78	0.84	0.71	2.45
*\checkmark*		*\checkmark*				0.74	0.88	0.76	0.90	0.76	2.78
*\checkmark*			*\checkmark*			0.73	0.92	0.74	0.92	0.74	2.47
*\checkmark*			*\checkmark*	*\checkmark*		0.74	0.95	0.75	0.95	0.78	2.68
*\checkmark*			*\checkmark*	*\checkmark*	*\checkmark*	0.88	0.93	0.94	0.93	0.94	2.01

**Table 2:** Statistical significance analysis of ablation components on the internal test set

Methods	mIoU	mPA	Precision	Recall	Dice	HD
w/o Combination	0.003	0.005	0.004	0.001	0.004	0.003
w/o Detect box	0.008	0.001	0.006	0.004	0.001	0.002
w/o Attention	0.008	0.007	0.005	0.002	0.001	0.005

**Fig. 4:**
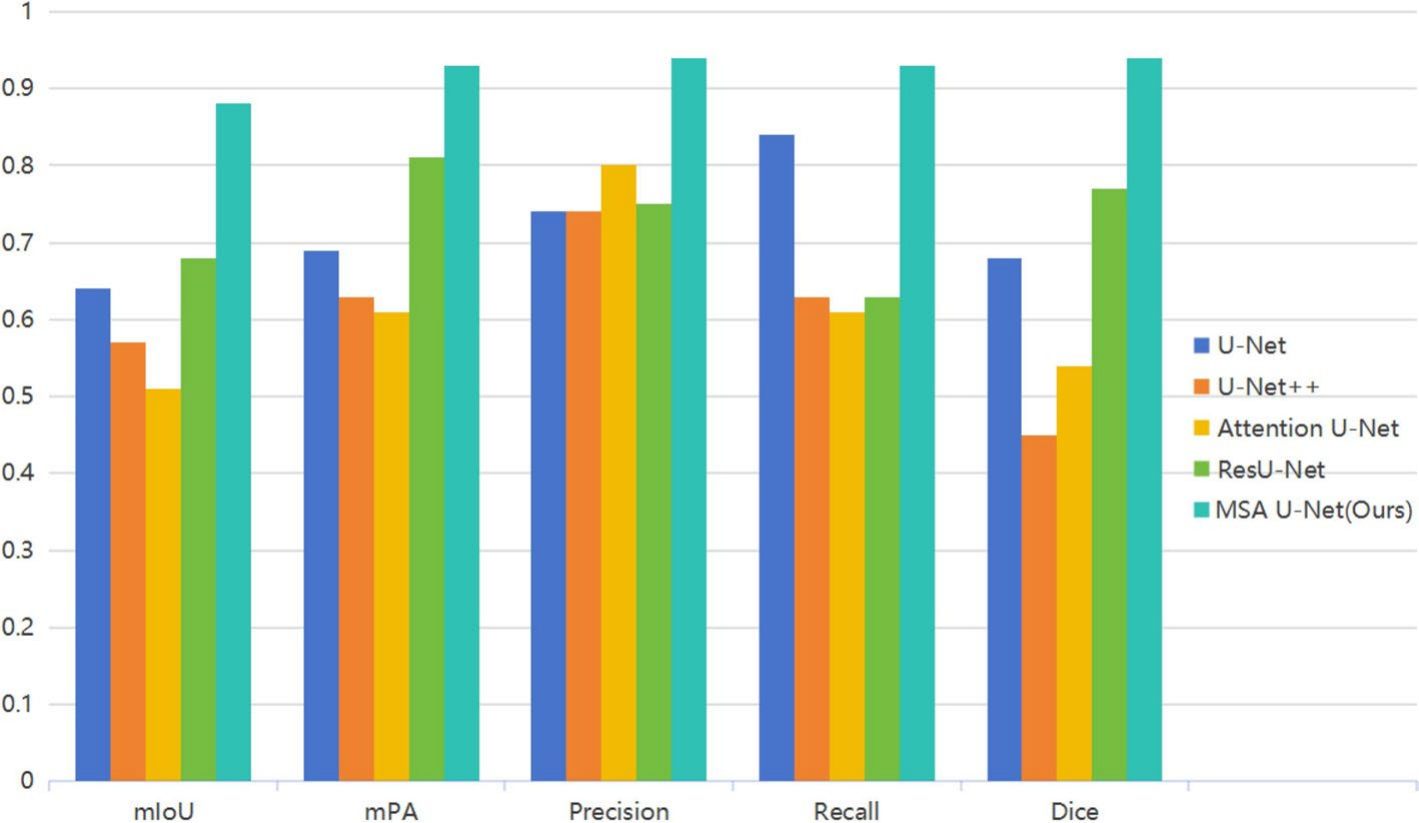
Quantitative comparison of segmentation models

Table 3 and Fig. 4 present a quantitative comparison of the segmentation performance of different models, including U-Net, U-Net++, Attention U-Net, ResU-Net, and the proposed MSA U-Net. Our method consistently achieves the best performance across all evaluation metrics, demonstrating its superior capability for LN segmentation in MRI images. Compared with the baseline U-Net, the proposed method improves the Dice coefficient from 0.68 to 0.94 and achieves a precision of 0.94 and a recall of 0.93, indicating both high segmentation accuracy and balanced detection performance. In contrast, although Attention U-Net attains a relatively high precision (0.80), its low recall (0.61) indicates that many LN regions are missed during segmentation. Furthermore, our method achieves the lowest Hausdorff Distance (HD) of 2.01, demonstrating its ability to generate more accurate and consistent LN boundaries.

The superior performance of the proposed method can be attributed to the complementary design of its key components. The multi-scale feature extraction module effectively captures both global contextual information and fine-grained local features, which are essential for accurately segmenting small and irregular LN structures. The CBSAM module further enhances discriminative feature representation by emphasizing informative regions while suppressing irrelevant background responses. In addition, the integration of the auxiliary detection head with the segmentation network provides more accurate LN localization and facilitates boundary refinement, thereby reducing both false-positive and false-negative predictions. Compared with ResU-Net (Dice = 0.77, HD = 2.35), the proposed method not only benefits from enhanced multi-scale feature representation but also achieves more precise boundary delineation through attention-guided feature refinement. These results demonstrate that the proposed modules work synergistically to improve segmentation accuracy and robustness, making MSA U-Net well suited for clinically relevant LN segmentation in uterine MRI images.

To further evaluate whether the observed performance improvements were statistically significant, a Wilcoxon signed-rank test was conducted using case-level Dice scores on the internal test set. As shown in Table 4, the proposed MSA-UNet achieved statistically significant improvements over all competing methods (*p* < 0.05), confirming that the performance gains were unlikely to be caused by random variation.

**Fig. 5:**
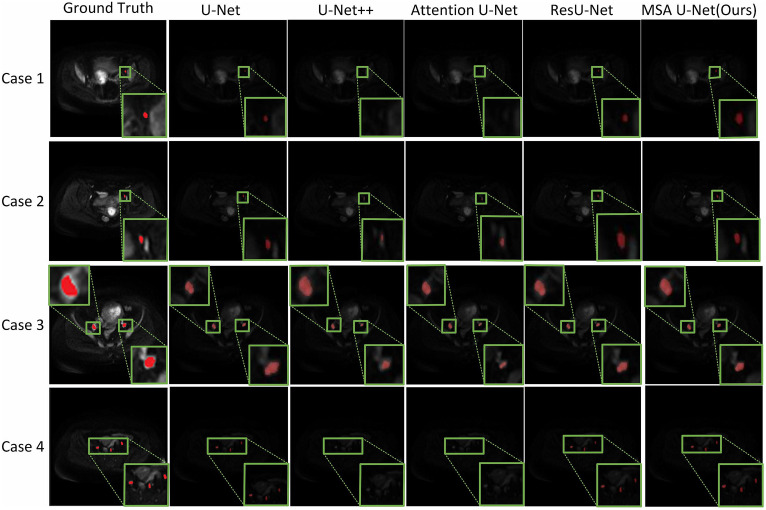
Segmentation results were obtained by different models

Figure 5 provides a visual comparison of the various models. The baseline U-Net and U-Net++ often exhibit incomplete segmentation or fail to capture small LN, particularly in Cases 1 and 2. While Attention U-Net achieves higher accuracy, it often under-segments LN, missing subtle boundary details. ResU-Net provides more continuous contours, but still suffers from false positives and blurred edges. In contrast, the segmentation masks generated by our MSA U-Net are visually closest to the ground truth, accurately capturing small and irregularly shaped LN while preserving clear and continuous boundaries. The zoomed-in view in Fig. 5 highlights our method’s ability to suppress background noise and enhance true lesion regions, reflecting the advantages of multi-scale feature fusion and the CBSAM module in improving localization and contour accuracy.

**Table 3:** Comparison of segmentation performance metrics among different models on the internal test set

Model	mIoU	mPA	Precision	Recall	Dice	HD
U-Net	0.64	0.69	0.74	0.84	0.68	2.56
U-Net++	0.57	0.63	0.74	0.63	0.45	10.54
Attention U-Net	0.51	0.61	0.80	0.61	0.54	22.10
ResU-Net	0.68	0.81	0.75	0.63	0.77	2.35
MSA U-Net(Ours)	0.88	0.93	0.94	0.93	0.94	2.01

**Table 4:** Statistical significance analysis based on Wilcoxon signed-rank test

Comparison	*p*-value
MSA U-Net vs U-Net	<0.001
MSA U-Net vs U-Net++	<0.001
MSA U-Net vs Attention U-Net	0.003
MSA U-Net vs ResU-Net	0.018

**Table 5:** Comparison of runtime performance and model complexity across different segmentation networks

Model	Params(M)	FLOPs(G)	Inference time(ms/image)	FPS
U-Net	24.89	452.31	18.53	53.98
Attention U-Net	25.29	464.40	16.14	61.95
ResUNet	25.67	475.93	15.76	63.45
MSA-UNet	109.09	1183	34.51	28.97

As shown in Table 5, the proposed MSA-UNet introduces additional computational complexity compared with conventional U-Net-based architectures due to the incorporation of multi-scale feature extraction modules, attention refinement blocks, and the auxiliary detection branch. Although the parameter count and computational cost increase accordingly, the model maintains practical inference efficiency, achieving an average inference time of 34.51 ms per image and a throughput of 28.97 FPS, which satisfies real-time clinical application requirements. Notably, the proposed CBSAM remains lightweight, introducing only 33.83k parameters while reducing computational cost by approximately 28.8% compared with the original CBAM (32.87k parameters). These results suggest that the additional complexity is primarily associated with the enhanced multi-scale representation and detection components, and is justified by the substantial improvement in segmentation performance and localization accuracy.

Table 6 presents the quantitative results of the proposed MSA U-Net under different pretraining strategies. Without pretraining, the model achieved an mIoU of 0.70 and a Dice coefficient of 0.67. Initializing the network with ResNet50 pretrained weights improved segmentation performance, increasing the Dice coefficient to 0.78 while reducing the HD from 3.54 to 2.92, indicating more accurate boundary delineation. Among all pretraining strategies, VGG pretraining achieved the best overall performance, yielding an mIoU of 0.88, mPA of 0.93, precision of 0.94, recall of 0.93, Dice coefficient of 0.94, and the lowest HD of 2.01. These results demonstrate that appropriate pretraining substantially enhances feature representation, leading to improved segmentation accuracy and more precise boundary localization for small LN structures.

Furthermore, the robustness and generalization capability of the proposed model were evaluated on both the internal and external test sets, as summarized in Table 7. On the internal test set, MSA U-Net achieved an mIoU of 0.88 and a Dice coefficient of 0.94. Despite domain shifts caused by differences in imaging protocols, noise characteristics, and annotation styles, the model maintained competitive performance on the external test set, achieving an mIoU of 0.68, mPA of 0.82, precision of 0.72, recall of 0.85, and a Dice coefficient of 0.76, while the HD increased to 3.84. The relatively high recall on the external dataset indicates that the proposed model remains sensitive to LN regions in previously unseen clinical data, whereas the moderate decrease in overall performance reflects the inherent challenges of cross-center generalization. Overall, these results demonstrate that the proposed MSA U-Net, together with the CBSAM module and an appropriate pretraining strategy, not only achieves high segmentation accuracy but also exhibits strong robustness and adaptability, highlighting its potential for deployment in multi-center clinical MRI applications.

**Table 6:** Performance comparison of MSA U-Net with different pretraining strategies on the internal test set

Model	mIoU	mPA	Precision	Recall	Dice	HD
MSA U-Net(no pretrain)	0.70	0.81	0.74	0.84	0.67	3.54
MSA U-Net+ResNet50(pretrain)	0.73	0.82	0.74	0.85	0.78	2.92
MSA U-Net+VGG(pretrain)	0.88	0.93	0.94	0.93	0.94	2.01

**Table 7:** Performance of MSA U-Net on internal and external test sets

Dataset	mIoU	mPA	Precision	Recall	Dice	HD
External	0.68	0.82	0.72	0.85	0.76	3.84
Internal	0.88	0.93	0.94	0.93	0.94	2.01

**Fig. 6:**
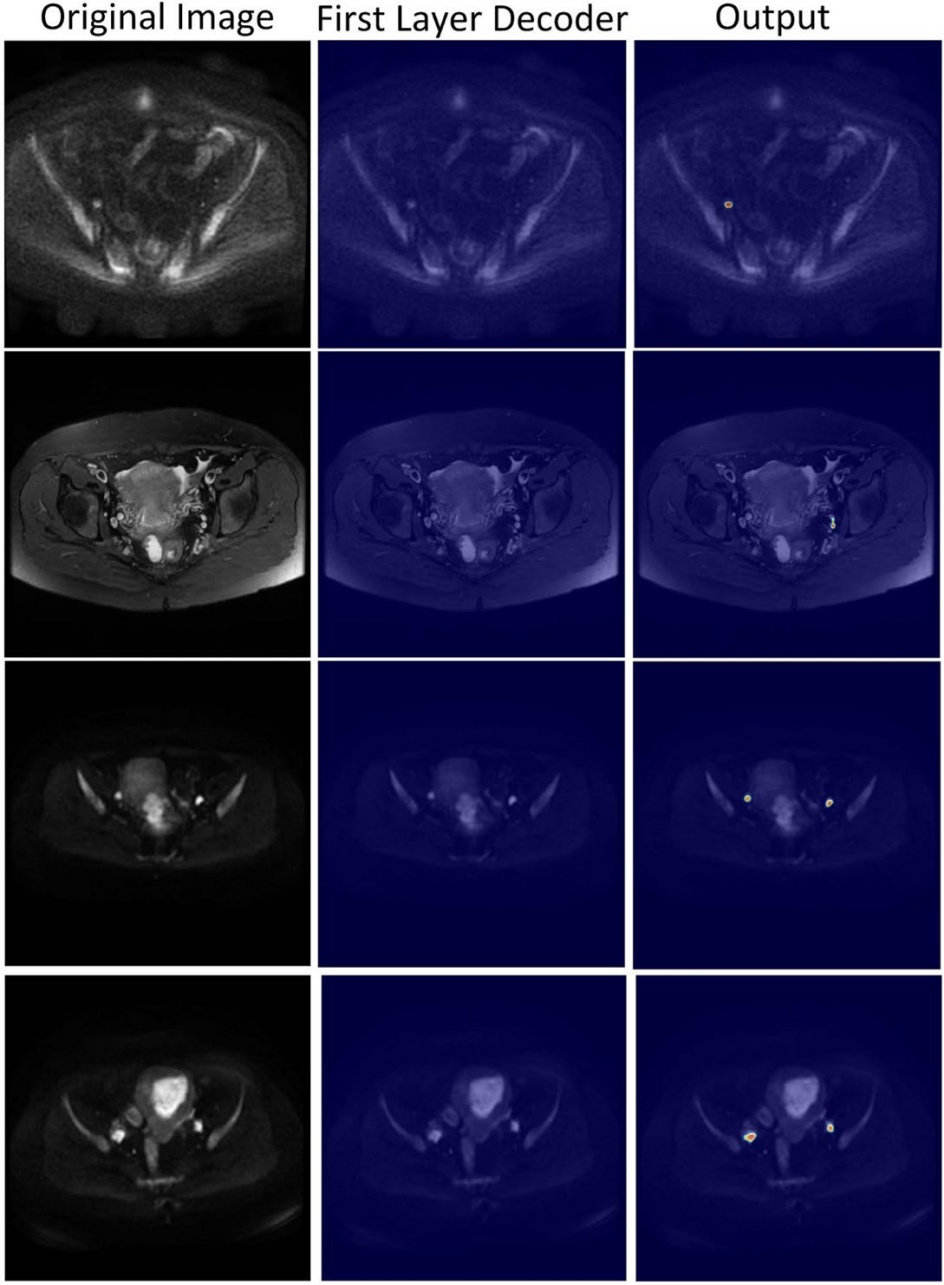
Visualization of feature activation maps from the first decoder layer and the final output

## Discussion

As shown in Table 1, the relatively low precision observed in some results reflects a trade-off commonly encountered in small-object medical image segmentation. In this study, the proposed MSA U-Net was designed to favor higher sensitivity, as reflected by the high recall values, in order to minimize missed LN detections. The false negatives are generally more critical than false positives in LN assessment, since missed suspicious nodes may directly affect staging and treatment planning. Although the improvements in some metrics appear moderate, the consistent increase in Dice coefficient and the reduction in HD indicate more accurate boundary delineation. For small and irregular LNs, precise contour definition is often more clinically relevant than coarse region overlap, as boundary errors may lead to inaccurate size estimation or misinterpretation of nodal involvement. Therefore, the observed improvements, particularly in HD, suggest that the proposed method can provide more reliable anatomical representations while maintaining high sensitivity.

The superior segmentation performance of MSA U-Net, as summarized in Table 3, can be understood through the interactions between its key architectural components. The incorporation of multi-scale feature extraction within the skip connections and parallel dilated convolutions at the bottleneck enables effective integration of global contextual information with fine-grained spatial details, which is particularly beneficial for segmenting small and irregular LNs. The attention mechanism further enhances feature discrimination by suppressing background responses and emphasizing lesion-relevant regions, while the auxiliary detection head provides coarse spatial guidance that facilitates boundary refinement in the decoder. In addition, the choice of pretraining strategy plays a critical role in determining segmentation performance. Table 6 provides a detailed comparison of the proposed MSA U-Net under different pretraining strategies. Compared with no pretraining and ResNet-based pretraining, the VGG-pretrained backbone yields more precise boundary delineation and higher recall, suggesting a stronger capacity for capturing low-level edge and texture cues that are essential for LN segmentation in MRI. These observations indicate that the performance gains of MSA U-Net arise from the alignment between multi-scale contextual modeling, targeted attention, detection-guided refinement, and modality-appropriate feature representations.

To further elucidate the role of the decoder in the proposed network, Fig. 6 visualizes the activation maps from the first decoder layer to the final output stage. In the initial decoder layer, activations are relatively diffuse, spanning broad anatomical regions and lacking precise LN localization, which is expected because early decoding stages integrate coarse semantic features from deeper layers with limited spatial resolution. As decoding progresses, the CBSAM mechanism effectively suppresses irrelevant background responses while selectively enhancing signals in lesion-relevant areas. By the final output stage, attention maps are sharply focused on the LN regions, accurately aligning with their true anatomical locations in the original MRI scans. This progressive focusing behavior highlights the decoder’s capacity to restore fine-grained spatial details, maintain semantic consistency, and improve the precision of small-object segmentation in complex pelvic MRI environments. These observations underscore the critical contribution of the decoder and the integrated attention mechanism in refining feature representations and ensuring accurate delineation of subtle anatomical structures.

**Fig. 7:**
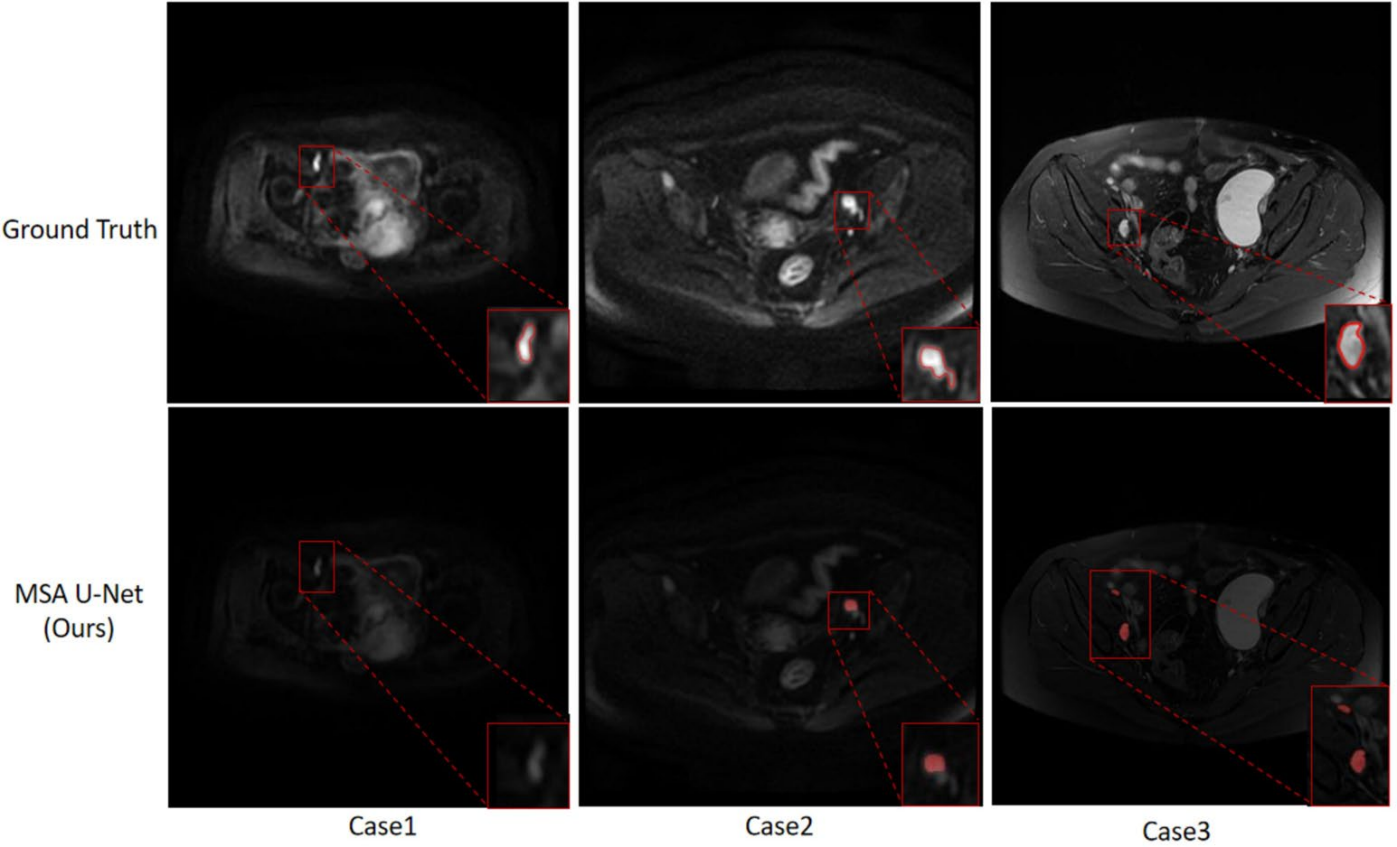
Representative failure cases of MSA U-Net. Case 1: missed segmentation of a LN. Case 2: under-segmentation caused by indistinct lesion boundaries. Case 3: over-segmentation due to confusion with surrounding tissues exhibiting similar MRI appearances

To better understand the limitations of the proposed model, Fig. 7 presents representative failure cases of MSA U-Net. Three common error patterns were observed. First, missed segmentation (Case 1) mainly occurred when the metastatic LN exhibited imaging characteristics similar to those of adjacent hyperintense structures. The limited discriminability between the lesion and surrounding tissues reduced the effectiveness of feature representation, causing the network to fail to distinguish the true LN from the background and resulting in a false-negative prediction. Second, under-segmentation (Case 2) was typically associated with indistinct or irregular LN boundaries. When the margins between the LN and surrounding soft tissues were poorly defined, the model tended to preserve only the most discriminative central region while omitting peripheral portions of the lesion. Third, over-segmentation (Case 3) was observed when neighboring anatomical structures exhibited imaging characteristics similar to those of LNs. In particular, blood vessels, bowel loops, inflammatory tissues, or adjacent soft-tissue structures occasionally produced signal patterns resembling metastatic LNs, leading the model to include non-LN regions in the predicted mask. Despite these challenging cases, the majority of LN regions were accurately delineated, indicating that the proposed framework remains robust under typical clinical conditions. Future work will focus on incorporating larger and more diverse datasets, as well as stronger anatomical priors and multi-modal information, to further reduce these error modes.

From a clinical perspective, achieving high segmentation accuracy for small anatomical structures such as pelvic LNs is essential for improving diagnostic precision, treatment planning, and follow-up monitoring in uterine cancer patients. The proposed model, with its balanced accuracy and efficiency, is well suited for time-sensitive applications such as intraoperative navigation and rapid pre-treatment assessment. Moreover, the reduced computational cost of CBSAM facilitates deployment in resource-constrained hospital environments, where high-end GPUs may not be available. Real-time performance also enables integration of the system into PACS for automated screening. The improved recall rates obtained with VGG pretraining may help reduce the risk of missed detections, which is particularly important in oncology applications where early identification of metastases significantly influences prognosis.

Despite the promising results, several limitations should be acknowledged.

First, although this study includes both an internal dataset and an external test set to assess generalizability, the external dataset is relatively small, comprising only 45 MRI images. Such a limited sample size may reduce the statistical power of the evaluation and limit the reliability of conclusions regarding cross-center robustness. Moreover, as most data originate from a single institution, potential bias cannot be fully excluded. Future work should incorporate larger, multi-center datasets with diverse MRI protocols to more comprehensively evaluate model robustness across clinical settings.

Second, although several measures were adopted to mitigate overfitting, including extensive data augmentation, patient-level data partitioning, early stopping, and independent external validation, the relatively limited sample size may still restrict the model’s generalization capability. Therefore, further validation on larger and more heterogeneous datasets is necessary before broader clinical deployment.

Third, while all annotations were reviewed and finalized through expert consensus, quantitative evaluation of interobserver variability was beyond the scope of the current study. Formal assessment of annotation consistency should therefore be incorporated in future studies to further validate the reliability of the reference annotations.

Fourth, segmentation performance was not separately analyzed according to LN size. Although lesion size may influence segmentation difficulty, no universally accepted MRI-based criterion currently exists for categorizing pelvic metastatic LNs into small-, medium-, and large-size groups in segmentation studies. In addition, the limited number of annotated LNs in our dataset would not support statistically reliable subgroup analysis. Future work will investigate size-stratified evaluation using larger datasets and standardized categorization criteria.

Beyond addressing these limitations, further exploration of lightweight hybrid CNN–Transformer architectures could yield additional performance gains without compromising real-time capability. Incorporating multi-modal imaging data, such as PET–MRI or contrast-enhanced MRI sequences, may also enhance the model’s ability to detect LNs with atypical appearances. Finally, explainability tools beyond activation maps, such as attention rollout or occlusion sensitivity analysis, could be adopted to provide deeper insights into the model’s decision-making process, thereby improving clinical interpretability and facilitating future clinical application.

## Conclusion

In this study, we proposed MSA U-Net, an attention-enhanced multi-scale network for the automated segmentation of uterine LNs in MRI scans. By integrating multi-scale feature fusion, a lightweight attention mechanism, and an auxiliary detection head, the model achieves state-of-the-art performance, with a Dice coefficient of 0.92 and an HD of 2.47. These advancements address key challenges in LN segmentation, including small-object detection and boundary ambiguity. The experimental results demonstrate the effectiveness of each proposed component, showing significant improvements over baseline methods. The ability of MSA U-Net to balance computational efficiency and segmentation accuracy makes it a promising tool for clinical applications, particularly for diagnosis and treatment planning. Future work will focus on expanding the dataset and further optimizing the model for real-time deployment in clinical workflows. Overall, this study contributes to the broader field of medical image analysis by providing a reliable and automated solution for LN segmentation, thereby supporting improved clinical decision-making and patient outcomes.

## Data Availability

Data will be made available from the corresponding author on reasonable request.

## References

[1] Zhao L, Wang T, Chen Y, Zhang X, Tang H, Lin F, et al. A novel framework for segmentation of small targets in medical images. Sci Rep. 2025;15(1):9924. 40121297 10.1038/s41598-025-94437-9 PMC11929788

[2] Liang D, Qiu J, Wang L, Yin X, Xing J, Yang Z, et al. Coronary angiography video segmentation method for assisting cardiovascular disease interventional treatment. BMC Med Imaging. 2020;20:1–8. 10.1186/s12880-020-00460-9 PMC7298947 32546137

[3] Zhang K, Zhang L, Pan H. UoloNet: based on multi-tasking enhanced small target medical segmentation model. Artif Intell Rev. 2024;57(2):31.

[4] Çiçek Ö, Abdulkadir A, Lienkamp SS, Brox T, Ronneberger O. 3D U-Net: learning dense volumetric segmentation from sparse annotation. In: Medical Image Computing and Computer-Assisted Intervention–MICCAI 2016: 19th International Conference, Athens, Greece, October 17-21, 2016, Proceedings, Part II 19. Springer; 2016. pp. 424–432.

[5] Badrinarayanan V, Kendall A, Cipolla R. SegNet: A deep convolutional encoder-decoder architecture for image segmentation. IEEE Trans Pattern Anal Mach Intell. 2017;39(12):2481–2495. 10.1109/TPAMI.2016.2644615 28060704

[6] Fang Y, Chen C, Yuan Y, Tong Ky. Selective feature aggregation network with area-boundary constraints for polyp segmentation. In: Medical Image Computing and Computer Assisted Intervention–MICCAI 2019: 22nd International Conference, Shenzhen, China, October 13–17, 2019, Proceedings, Part I 22. Springer; 2019. pp. 302–310.

[7] He K, Zhang X, Ren S, Sun J. Deep residual learning for image recognition. In: Proceedings of the IEEE Conference on Computer Vision and Pattern Recognition. IEEE; 2016. p. 770–778.

[8] Huang G, Liu Z, van der Maaten L, Weinberger KQ. Densely connected convolutional networks. In: Proceedings of the IEEE Conference on Computer Vision and Pattern Recognition. IEEE; 2017. p. 4700–4708.

[9] Tan M, Le Q. EfficientNet: Rethinking model scaling for convolutional neural networks. In: Proceedings of the 36th International Conference on Machine Learning. PMLR; 2019. p. 6105–6114.

[10] Gu Z, Cheng J, Fu H, Zhou K, Hao H, Zhao Y, et al. Ce-net: context encoder network for 2d medical image segmentation. IEEE Trans Med Imaging. 2019;38(10):2281–2292. 30843824 10.1109/TMI.2019.2903562

[11] Fu H, Cheng J, Xu Y, Wong DWK, Liu J, Cao X. Joint optic disc and cup segmentation based on multi-label deep network and polar transformation. IEEE Trans Med Imaging. 2018;37(7):1597–1605. 29969410 10.1109/TMI.2018.2791488

[12] Liao W, Luo X, Li L, Xu J, He Y, Huang H, et al. Automatic cervical lymph nodes detection and segmentation in heterogeneous computed tomography images using deep transfer learning. Sci Rep. 2025;15(1):4250. 39905029 10.1038/s41598-024-84804-3 PMC11794882

[13] Roth HR, Lu L, Seff A, Cherry KM, Hoffman J, Wang S, et al. A new 2.5D representation for lymph node detection using random sets of deep convolutional neural network observations. In: Proceedings of Medical Image Computing and Computer-Assisted Intervention. Springer; 2014. p. 520–527. 10.1007/978-3-319-10404-1_65 PMC4295635 25333158

[14] Zhao X, Xie P, Wang M, Li W, Pickhardt PJ, Xia W, et al. Deep learning–based fully automated detection and segmentation of lymph nodes on multiparametric MRI for rectal cancer: A multicentre study. EBioMedicine. 2020;56:102780. 10.1016/j.ebiom.2020.102780 PMC7276514 32512507

[15] Rinneburger M, Carolus H, Iuga AI, Weisthoff M, Lennartz S, Hokamp NG, et al. Automated localization and segmentation of cervical lymph nodes on contrast-enhanced CT using a 3D foveal fully convolutional neural network. Eur Radiol Exp. 2023;7(1):45. 37505296 10.1186/s41747-023-00360-x PMC10382409

[16] Cao Y, Feng J, Wang C, Yang F, Wang X, Xu J, et al. LNAS: A clinically applicable deep-learning system for mediastinal enlarged lymph nodes segmentation and station mapping without regard to the pathogenesis using unenhanced CT images. Radiol Med (Torino). 2024;129(2):229–238. 38108979 10.1007/s11547-023-01747-x

[17] Zhou Z, Siddiquee MMR, Tajbakhsh N, Liang J. UNet++: redesigning skip connections to exploit multiscale features in image segmentation. IEEE Trans Med Imaging. 2019;39(6):1856–1867. 10.1109/TMI.2019.2959609 PMC7357299 31841402

[18] Huang H, Lin L, Tong R, Hu H, Zhang Q, Iwamoto Y, et al. UNet 3+: A Full-Scale Connected UNet for Medical Image Segmentation, In: 2020 IEEE International Conference on Acoustics, Speech and Signal Processing (ICASSP). Barcelona, Spain; 2020. p. 1055–1059.

[19] Alom MZ, Hasan M, Yakopcic C, Taha TM, Asari VK. Recurrent residual convolutional neural network based on U-Net (R2U-Net) for medical image segmentation. 2018. arXiv preprint arXiv:1802.06955.

[20] Ibtehaz N, Rahman MS. MultiResUNet: Rethinking the U-Net architecture for multimodal biomedical image segmentation. Neural Netw. 2020;121:74–87. 31536901 10.1016/j.neunet.2019.08.025

[21] Azad R, Asadi-Aghbolaghi M, Fathy M, Escalera S. Bi-directional ConvLSTM U-Net with densley connected convolutions. In: Proceedings of the IEEE/CVF International Conference on Computer Vision Workshops. IEEE; 2019. p. 1–10.

[22] Chen LC, Zhu Y, Papandreou G, Schroff F, Adam H. Encoder-decoder with atrous separable convolution for semantic image segmentation. In: Proceedings of the European Conference on Computer Vision. Springer; 2018. p. 801–818.

[23] Xie L, Cai W, Gao Y. Dmcgnet: A novel network for medical image segmentation with dense self-mimic and channel grouping mechanism. IEEE J Biomed Health Inform. 2022;26(10):5013–5024. 35939480 10.1109/JBHI.2022.3192277

[24] Liu Y, Zhou J, Liu L, Zhan Z, Hu Y, Fu Y, et al. FCP-Net: A feature-compression-pyramid network guided by game-theoretic interactions for medical image segmentation. IEEE Transactions on Medical Imaging. 2022;41(6):1482–1496. 10.1109/TMI.2021.3140120 34982679

[25] Song E, Zhan B, Liu H, Cetinkaya C, Hung CC. NMNet: learning multi-level semantic information from scale extension domain for improved medical image segmentation. Biomed Signal Process Control. 2023;83:104651.

[26] Agarwal R, Ghosal P, Sadhu AK, Murmu N, Nandi D. Multi-scale dual-channel feature embedding decoder for biomedical image segmentation. Comput Methods Programs Biomed. 2024;257:108464. 39447437 10.1016/j.cmpb.2024.108464

[27] Gong L, Liu L, Liu G, Bai H, Wang D, Wang H. PLNM-YOLO: pelvic lymph node metastasis detection algorithm in gynaecological tumours based on edge-aware and multi-scale feature fusion. Biomed Signal Process Control. 2026;112:108358.

[28] Datta A, Sarkar S, Sharma AL, Ghosal P. Multimodal exponential moving average-guided representation for integrated tumor-segmentation and survival prediction. Eng Appl Artif Intell. 2026;167:113868.

[29] Oktay O, Schlemper J, Folgoc LL, Lee M, Heinrich M, Misawa K. Attention u-net: Learning where to look for the pancreas. 2018. arXiv preprint. arXiv:1804.03999.

[30] Gu R, Wang G, Song T, Huang R, Aertsen M, Deprest J, et al. CA-Net: Comprehensive attention convolutional neural networks for explainable medical image segmentation. IEEE Trans Med Imaging. 2020;40(2):699–711. 10.1109/TMI.2020.3035253 PMC7611411 33136540

[31] Wang M, Zhu W, Shi F, Su J, Chen H, Yu K, et al. MsTGANet: Automatic drusen segmentation from retinal OCT images. IEEE Trans Med Imaging. 2021;41(2):394–406. 10.1109/TMI.2021.3112716 34520349

[32] Zhan B, Song E, Liu H, Gong Z, Ma G, Hung CC. CFNet: a medical image segmentation method using the multi-view attention mechanism and adaptive fusion strategy. Biomed Signal Process Control. 2023;79:104112.

[33] Wang T, Liu J, Tang J. A cross-scale attention-based U-net for breast ultrasound image segmentation. J Imaging Inform Med. 2025;38(5):2851–2864. 39838227 10.1007/s10278-025-01392-y PMC12572516

[34] Liu X, Pan J, Li X, Wei X, Liu Z, Pan Z, et al. Attention based cross-domain synthesis and segmentation from unpaired medical images. IEEE Trans Emerg Top Comput Intel. 2023;8(1):917–929.

[35] Lin Y, Li X, Zhang Y, Tang J. Attention-based efficient classification for 3D MRI image of Alzheimer's disease. In: Proceedings of the 2023 6th International Conference on Sensors, Signal and Image Processing. Association for Computing Machinery; 2023. p. 34–39.

[36] Woo S, Park J, Lee JY, Kweon IS. CBAM: Convolutional block attention module. In: Proceedings of the European Conference on Computer Vision. Springer; 2018. p. 3–19.

